# Probiotic *Lactobacillus casei*: Effective for Managing Childhood Diarrhea by Altering Gut Microbiota and Attenuating Fecal Inflammatory Markers

**DOI:** 10.3390/nu11051150

**Published:** 2019-05-23

**Authors:** Hung-Hsiang Lai, Cheng-Hsun Chiu, Man-Shan Kong, Chee-Jen Chang, Chien-Chang Chen

**Affiliations:** 1 Division of Gastroenterology, Department of Pediatrics, Chang Gung Memorial Hospital, Chang Gung University College of Medicine, Taoyuan 33303, Taiwan; b101095062@tmu.edu.tw (H.-H.L.); kongchi@cgmh.org.tw (M.-S.K.); 2 Division of Infectious Disease, Department of Pediatrics, Chang Gung Memorial Hospital, Chang Gung University College of Medicine, Taoyuan 33303, Taiwan; chchiu@cgmh.org.tw; 3 Graduate Institute of Clinical Medical Sciences, Clinical Informatics and Medical Statistics Research Center, Chang Gung University College of Medicine, Taoyuan 33303, Taiwan; cjchang@mail.cgu.edu.tw

**Keywords:** probiotics, *Lactobacillus casei* variety *rhamnosus* (Lc), microbiota, immunoglobulin A (IgA), lactoferrin, calprotectin

## Abstract

Background: Acute diarrhea is a major cause of childhood morbidity and an economic burden for families. The aim of this study is to explore the effect of probiotics on clinical symptoms, intestinal microbiota, and inflammatory markers during childhood diarrhea. Methods: Children (*n* = 81) aged six months to six years (mean age 2.31 years) hospitalized for acute diarrhea were randomized to receive probiotics (*Lactobacillus casei* variety *rhamnosus*; *n* = 42) or no probiotics (*n* = 39) orally twice daily for seven days. Feces samples were also collected to evaluate microbial content using a traditional agar plate and next-generation sequencing. Immunoglobulin A (IgA), lactoferrin, and calprotectin were determined by enzyme-linked immunosorbent assay (ELISA) and compared in different groups. Other clinical symptoms or signs, including fever, vomiting, diarrhea, abdominal pain, bloated abdomen, daily intake, appetite, and body weight were also assessed. Results: Data were collected from 81 individuals across three different time points. Total fecal IgA levels in fecal extracts of the probiotics group were higher than those in the control group, reaching statistical significance (*p <* 0.05). Concentrations of fecal lactoferrin and calprotectin were significantly downregulated in patients with probiotic *Lactobacillus casei* variety *rhamnosus* (Lc) consumption compared to those of the control (*p <* 0.05). Probiotic Lc administration may be beneficial for gut-microbiota modulation, as shown by the data collected at one week after enrollment. Counts of *Bifidobacteria* and *Lactobacillus* species were elevated in stool culture of the probiotic group. Appetite and oral intake, body-weight gain, abdominal pain, bloating, as well as bowel habits (diarrhea) were much better in children receiving probiotics compared with those in the control group. Conclusion: Fecal IgA increased during acute diarrhea under Lc treatment; in contrast, fecal lactoferrin and calprotectin were downregulated during acute diarrhea under Lc treatment. Probiotic Lc may be a useful supplement for application in children during acute diarrhea to reduce clinical severity and intestinal inflammatory reaction.

## 1. Introduction

Acute diarrhea caused by pathogens may induce gastroenteritis, bloody stool, or severe intra-abdominal infections that establish disease and increase the economic burden, especially among infantile and childhood populations.

Probiotic *Lactobacillus casei* can reduce the incidence of diarrhea and its duration in children [1,2]. Moreover, the incidence was reduced among healthy young adults [3] consuming yogurt containing *Lactobacillus casei*. The mechanism by which probiotic bacteria, which inhabit the gastrointestinal tracts of healthy individuals, exert their beneficial effects is unclear. They may do so by enhancing host immunity, inhibiting bacterial epithelial and mucosal adherence, inhibiting epithelial invasion, and/or producing antimicrobial substances [4,5].

Secretory IgA plays an important role in protection against infections caused by entero pathogens in both human and animal models [6]. IgA is enhanced by probiotics as a reaction related to the mucosal immunity of the host intestine. Intestinal micro-organisms may play an important role in the development of the acquired immune system of infants, especially in the development of mucosal immunity and the production of endogenous IgA [7].

Lactoferrin is a major whey protein, an 80 kDa iron-binding glycoprotein produced by many exocrine glands with a major constituent in the secondary granules of neutrophilic leukocytes. The presence of lactoferrin in body fluids, including intestinal lumen, is proportional to the flux of neutrophils and its assessment can provide a reliable biomarker for inflammation. Neutrophils have been shown to be involved in the perpetuation of inflammation in the gut in acute infection caused by bacterial pathogens as well as in inflammatory bowel disease (IBD) [8,9,10]. Guerrant et al. confirmed increased fecal lactoferrin in 96% (25/26) of samples from patients with bacteria intestinal infection and concluded that fecal lactoferrin is a useful marker for fecal leukocytes [11].

Calprotectin is a heterodimer of two calcium-binding proteins, S100A8 and S100A9. Both subunits are found at increased levels in the colon. Calprotectin is a 36 kDa calcium- and zinc-binding protein, and constitutes approximately 60% of soluble cytosolic proteins in neutrophilic granulocytes. Therefore, calprotectin is a marker of neutrophil influx and is elevated in a number of inflammatory conditions [12,13]. Elevated calprotectin levels are a good predictor of inflammation, as well as being associated with standard inflammatory markers (e.g., serum C-reactive protein (CRP)). Fecal calprotectin is emerging as a useful marker to quantify mucosal inflammation, not in the least because it appears to be stable in feces, which can be obtained by noninvasive means [14].

Our study sought to determine whether probiotics (*Lactobacillus casei*) inhibited gastrointestinal infection and reduced the associated inflammatory response. The bacterial counts and inflammatory parameters in fecal samples of acute childhood diarrhea were investigated. We assessed the effects of probiotics in the digestive system and fecal inflammatory markers of children with acute diarrhea.

## 2. Patients and Methods

### 2.1. Study Design

This prospective, randomized, case-controlled study analyzed children enrolled in Chang Gung Children’s Hospital located in Northern Taiwan. The study protocol was approved by the Institutional Review Board of Chang Gung Memorial Hospital, and the study was registered at ClinicalTrials.gov (NCT 03856138). Informed consent was obtained from at least one parent or legal guardian prior to study inclusion. The study was performed in accordance with the Declaration of Helsinki. The study protocol was approved by the Institutional Review Board of Chang Gung Memorial Hospital, Taiwan. There are no conflicts of interest. Trial registration number: ClinicalTrials.gov identifier: NCT03856138.

### 2.2. Patients

Subjects were divided into 2 groups (*Lactobacillus casei* variety *rhamnosus* treatment and control). Enrollment was carried out between December 2015 and February 2018. Diarrhea was defined as 3 or more outputs of loose or liquid stools per day. Inclusion criteria were aged 6 months to 6 years, and hospitalized children with suspicious infectious diarrhea of less than 72 h. Exclusion criteria were severe abdominal distension with risk of bowel perforation, immunodeficiency, risk for sepsis, underlying disorder with inflammatory bowel disease, history with surgical operation of the gastrointestinal tract, probiotic use in the 1 week preceding starting therapy, and antibiotics use in the hospital course.

Diet and probiotic micro-organisms: The preparation contained lyophilized powder *Lactobacillus casei* species. Each probiotic capsule contained 2 × 10^8^ colony-forming units (CFU)/250mg of *Lactobacillus casei* variety *rhamnosus* (Lcr35^®^) in lyophilized powder form. The test capsule was commercially available (Probionov, Aurillac, France), and contained freeze drying bacteria (Lcr35^®^), the microbes of which were cultured in Man, Rogosa, Sharpe (MRS) broth, suspended in a lyophilization medium, subjected to freeze-drying, and then stored properly in a capsule. Each participant in the probiotic group consumed the lyophilized powder in capsule form twice daily (total cells: 4 × 10^8^ CFUs/day) for 7 days over the course of the study. Both the probiotic and control groups were supplied by intravenous fluid hydration. Some clinical parameters were evaluated according to the following: body-weight change, appetite and daily intake, bloating or abdominal distension, abdominal or colic pain, diarrhea or fecal consistency, constipation, fever, and vomiting, which were also assessed. The appetite/intake score was evaluated by the parents or family of participants: 0, no oral intake; 1, little oral intake; 2, oral intake less than one-third of daily food; 3, oral intake more than one-third but less than two-thirds of daily food; 4, oral intake more than two-thirds of daily food; 5, very satisfied with oral intake.

### 2.3. Bacterial Culture and Probiotic-Bacteria Count

To assess the colonization of intestinal bacteria, fecal samples were collected from each patient on Days 0 (the day when patients were enrolled), 3, and 7, after probiotic or placebo treatment. The fecal specimens were weighed, homogenized, and serially diluted and plated on selective MacConkey agar for analysis of Gram-negative bacteria. After overnight incubation at 37 °C, bacteria colonies were counted. Bifidobacterium iodoacetate medium (BIM) agar plates were used to plate and analyze fecal *Bifidobacterium* counts. In order to assess the colonization effect of the *Lactobacillus* strain, fecal samples were also plated on deMan, Rogosa, and Sharpe agar (MRS; Difco), as well as Rogosa SL agar plates (Difco). In order to explore the total anaerobic bacteria counts, the fecal samples were also plated on anaerobic blood agar base plate (CDC-BAP) agar. After 48 to 72 h of anaerobic incubation at 37 °C, bacteria colonies could be confirmed with regard to colonization. Fecal bacteria count was expressed as log_10_ CFU/g feces.

### 2.4. Taxonomy-Based and Statistical Analyses

Approximately 0.2 g of a wet-sludge sample pellet was used for DNA extraction with a QIAamp^®^ Fast DNA Stool Mini Kit (Hilden, Germany), according to the manufacturer’s instructions. The extracted genomic DNA samples were examined by 1.5% agarose gel electrophoresis. Then, they were stored at −20 °C until use.

#### 2.4.1. 16 S rRNA Amplification

For each fecal sample, a 50 μL polymerase chain reaction (PCR) mix was prepared containing 25 ng DNA template, 5X KAPA HiFi Buffer, 10 mM KAPA dNTPMix, 1 U/μL KAPA HiFi DNA Polymerase (KAPA Biosystem, Boston, United States), and 0.3 μM of each primer (Tri-I, New Taipei, Taiwan). PCR reaction conditions consisted of an initial 95 °C for 3 min followed by 15–25 cycles of 98 °C for 20 sec, 45 °C for 15 sec, and 72 °C for 15 sec, and a final extension of 72 °C for 1 min. The PCR products were purified with a QIAquick PCR purification kit (Qiagen, Hilden, Germany), quantified using a Qubit^®^ dsDNA HS assay kit (Invitrogen, Grand Island, NY, USA) on a Qubit^®^ 2.0 Fluorometer (Invitrogen, Grand Island, NY, USA). PCR products were sequenced on an illumina sequencing platform according to the instructions of recommended Miseq procedures.

#### 2.4.2. Library Construction and Illumina MiSeq Sequencing

The paired-end library was constructed with Ovation^®^ Ultralow DR Multiplex System 1–96 (NuGen, San Carlos, CA, USA), all according to the respective manufacturers’ instructions. Library concentration and quality were assessed on a Bioanalyzer 2100 (Agilent, Palo Alto, CA, USA) using a DNA 1000 lab chip (Agilent, Palo Alto, CA, USA). Then, 16S amplicon libraries were sequenced 2 × 301 + 8 bp (index) by using a Miseq Reagent kit v3 (600 cycles) on an Illumina MiSeq system following the manufacturer’s instructions.

#### 2.4.3. Data-Processing Pipeline

The sequencing reads were initially demultiplexed using a MiSeq Reporter v2.6 according to the sample barcodes. The resulting pairs of reads from each sample were merged to obtain longer reads (460 ± 50 bp) to improve taxonomy classification using FLASH (V1.2.11) [15]. Only samples with merged reads ≥100,000 were retained for subsequent analysis. Low-quality reads with q-value <20 were filtered by the split_libraries_fastq.py script of QIIME (version 1.9.1) [16]. Only sequence tags with length >400 bp were retained for subsequent analysis. Operational taxonomic units (OTUs) were clustered at 97% sequence similarity using USEARCH (v9.2.64) [17] against the Greengenes 16S rRNA database (13_8 release), and the final taxonomic assignment was performed using an RDP classifier [18]. Clustal Omega software was used to construct phylogenetic trees from representative OUT sequences.

### 2.5. Fecal Immunoglobulin Assay

Fecal samples were collected during the treatment period. IgA levels were measured on homogenized fecal samples. Immuno II plates were coated with goat antihuman IgA (1μg/mL) and incubated for 60 min at room temperature. After blocking and washing, the plates were incubated with diluted fecal supernatant samples and standard human reference serum IgA Calibrator (Bethyl Laboratories, Inc). Total IgA was determined using a goat antihuman IgA-HRP conjugate. The reaction was developed with tetramethylbenzidine (TMB; Zymed Labs.) and read at 450 nm. OD values were converted to ng/g feces of total IgA by comparison with a standard curve developed with antihuman IgA.

### 2.6. Measurement of Inflammatory Mediators in the Intestine

Fecal samples of these patients were collected at 3 different time points, including the initial stage of acute diarrhea (the day when patients enrolled), 3 days later, and 7 days later. Series follow-up of fecal lactoferrin and calprotectin were measured by ELISA. Fecal extracts were prepared by mixing stool (1 g of feces in 4 mL of buffer) with phosphate-buffered saline (PBS) containing ethylenediaminetetraacetic acid (EDTA, 0.05 M), the protease inhibitor soybean trypsin (100 μg/mL), and phenylmethylsufonyl fluoride (10 mM). Fecal extracts were frozen in aliquots at −70 °C.

Lactoferrin assay: Stool samples were prepared and analyzed for lactoferrin according to the manufacturer’s instructions (AssayMax Human Lactoferrin ELISA Kit, St. Charles, MO, USA). This assay employs a quantitative sandwich enzyme immunoassay technique that measures lactoferrin in 4 h. A polyclonal antibody specific for lactoferrin was precoated onto a microplate. Lactoferrin in standards and samples was sandwiched by the immobilized antibody and a biotinylated polyclonal antibody specific for lactoferrin, which is recognized by a streptavidin–peroxidase conjugate. Absorbance was read at OD 450 nm. Lactoferrin was expressed as μg/g feces.

Calprotectin assay: Stool samples were prepared and analyzed for calprotectin according to the manufacturer’s instructions (*PhiCal* Calprotectin ELISA Kit, Immundiagnostik, Bensheim, Germany). Stool was collected in plastic containers and sent to the laboratory by mail either the same or the next day. The weight of each sample (100–500 mg) was measured, and an extraction buffer containing citrate was added in a weight/volume ratio of 1:50. The samples were mixed by vortex mixer for 30 s and homogenized for 25 min. One milliliter of the homogenate was transferred to a tube and centrifuged for 20 min at 10,000× *g*. Finally, the supernatant was collected and frozen at –20 °C.The supernatants were thawed, and calprotectin was analyzed with the quantitative calprotectin ELISA and read at OD 450 nm. Calprotectin was expressed as μg/g feces [19].

### 2.7. Statistical Analysis

Comparison between groups was performed with the *t*-test or the Mann–Whitney U test, the [chi]^2^ test, and ANOVA for repeated measures. Analyses were performed on the intention to treat the population. To explore time, treatment, and their interaction, a 2-factor ANOVA for repeated measures containing 3 time points and 2 groups, and the Tukey–Kramer post hoc test were used to analyze the data after verifying the normal distribution of the dataset. A significance level of 0.05 was used, and the statistical tests were two-tailed. GraphPad Software Prism 3.03 (GraphPad Software, Inc., San Diego, CA, USA) and SPSS Software, version 18.0 (SPSS Inc., Chicago, IL, USA), were used for statistical analysis.

## 3. Results

### 3.1. Subject Characteristics

A total of eighty-one children were enrolled in the study. All patients had acute diarrhea less than three days before enrollment. Study completers, which consisted of children (*n* = 81) aged six to 72 months (mean age 2.31 years) hospitalized for acute diarrhea, were randomized to receive probiotics (*Lactobacillus casei* variety *rhamnosus*) (*n* = 42) orally twice daily for seven days. Both groups were similar in sex, age, body weight, white-blood-cell (WBC) counts, fever, vomiting, as well as the duration of diarrhea before intervention (Table 1).

### 3.2. Parameters Related to Digestive System

Appetite and oral intake were much better in children receiving probiotics compared with those in the control group on Days 3, 7, and 14 (Table 2). Bloating or abdominal distension during treatment were also less in children receiving probiotics compared with the control group. Episodes of abdominal pain or colic during treatment were also fewer in children receiving probiotics compared with the control group on Days 3, 7, and 14. Diarrhea after the start of treatment was significantly reduced (*p* < 0.05) in children receiving probiotics compared with those in the control group on Days 3, 7, and 14. Duration of fever (>38 °C) in hours and episodes of vomiting were compared between probiotic and control groups during treatment; no significant finding was observed.

### 3.3. Fecal Bacterial Counts

We collected fecal samples on Days 0, 3, and 7 of the course. As shown in Table 3, compared to the control group, the total major probiotic population (*Lactobacillus* and *Bifidobacterium* species) in the probiotic group gradually increased to higher counts by recovering the bacterial culture of fecal specimens. During the consumption period, *Lactobacillus* bacterial counts of stool in the treatment (probiotic) group were 8.6 (8.0–9.4) log_10_ CFU/g feces on Day 3, 9.1 (8.3–9.8) log_10_CFU/g feces on Day 7, and *Bifidobacterium* bacteria counts were 9.7 (9.1–10.4) log_10_CFU/g feces on Day 7, which were higher than those in the control group, *p* < 0.05. The above results suggest that administration of probiotics (*Lactobacillus casei* variety *rhamnosus*) restores the major probiotic population in the intestinal tract during acute diarrhea.

There was no significant difference in anaerobic bacteria counts between the probiotic and placebo groups. A similar tendency was observed for the Gram-negative bacteria in the intestinal tract, but counts gradually decreased on Days 3 and 7 without reaching statistical significance. The above results suggest that probiotics may competitively inhibit Gram-negative bacteria in the intestinal tract, but have no remarkable effect on anaerobic bacteria populations.

### 3.4. Microbiota Analysis

We also performed next-generation sequencing for a gut-microbiota survey. Fecal samples collected at a week after enrollment in this study were used for gut-microbiota analysis.

Analysis of alpha diversity with the Shannon index revealed lower diversity of total microbiota for the children with a probiotic supplement (Shannon index 4.18 vs. 5.25, respectively, *p* = 0.03). Children with the probiotic supplement also presented significantly lower overall gut-microbiota richness compared with the control (mean Chao1 richness estimator: 163.65 vs. 217.42, respectively; *p* = 0.04).

### 3.5. Effects at the Phylum Level

**Figure 1:**
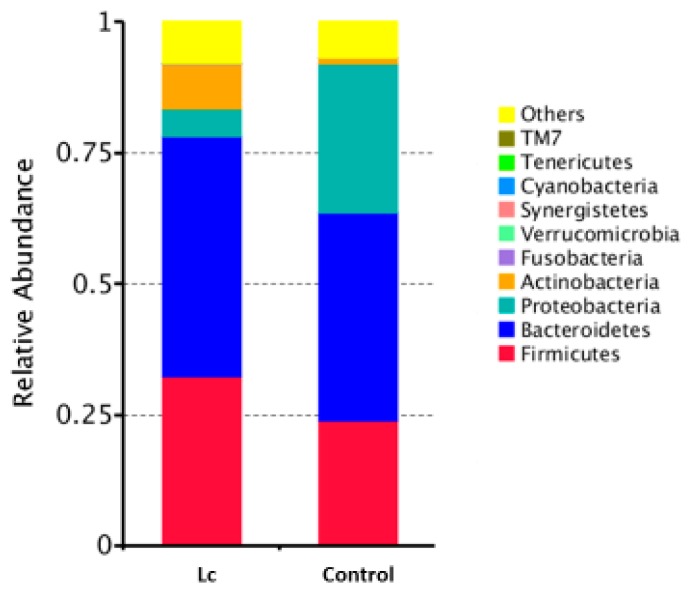
Differences in gut microbial composition between children with the probiotic *Lactobacillus casei* variety *rhamnosus* (Lc) supplement and the control group. Fecal samples of patients were collected at one week after enrollment in this study. Each color in this bar graph represents the corresponding taxon group at the phylum level in the legend.

At the phylum level, the mean relative abundance of Firmicutes, Actinobacteria, and Bacteroidetes was higher in children with the probiotic supplement compared to the control. The mean relative abundance of Proteobacteria was lower in children with the probiotic supplement compared to the control (Figure 1). There were no significant differences in the abundances of Fusobacteria and Verrucomicrobia between children with and without the probiotic supplement.

### 3.6. Alterations at Order Levels

**Figure 2:**
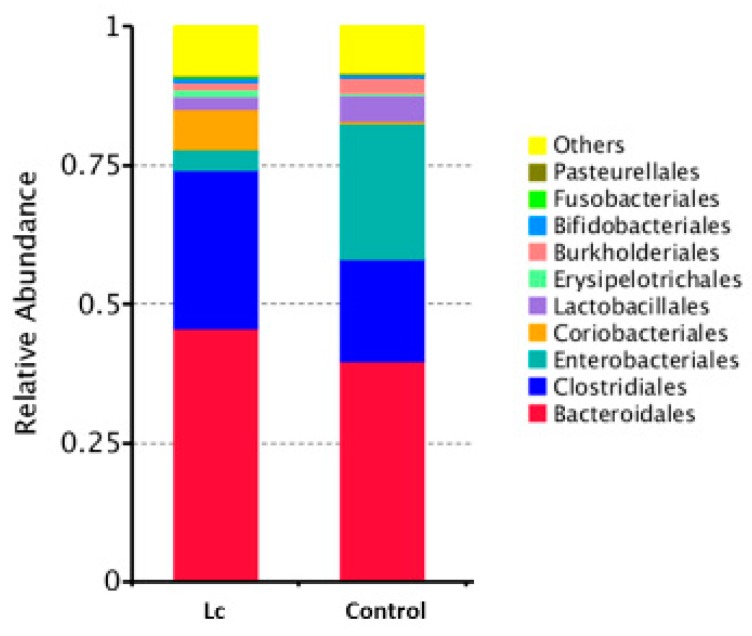
Differences in gut microbial composition between children with the probiotic *Lactobacillus casei* variety *rhamnosus* (Lc) supplement and the control group. Fecal samples of patients were collected at one week after enrollment in this study. Each color in this bar graph represents the corresponding taxon group at the order level in the legend.

At the order level, the mean relative abundance of Bacteroidales, Clostridiales, and Coriobacteriales was higher in the children with the probiotic supplement compared to the control group. The mean relative abundance of Enterobacteriales was lower in the children with the probiotic supplement compared to the control. In the case of children, the probiotic supplement may inhibit Enterobacteriales but enhance the relative abundance of Clostridiales (Figure 2).

### 3.7. Alterations at the Family and Genus Levels

**Figure 3:**
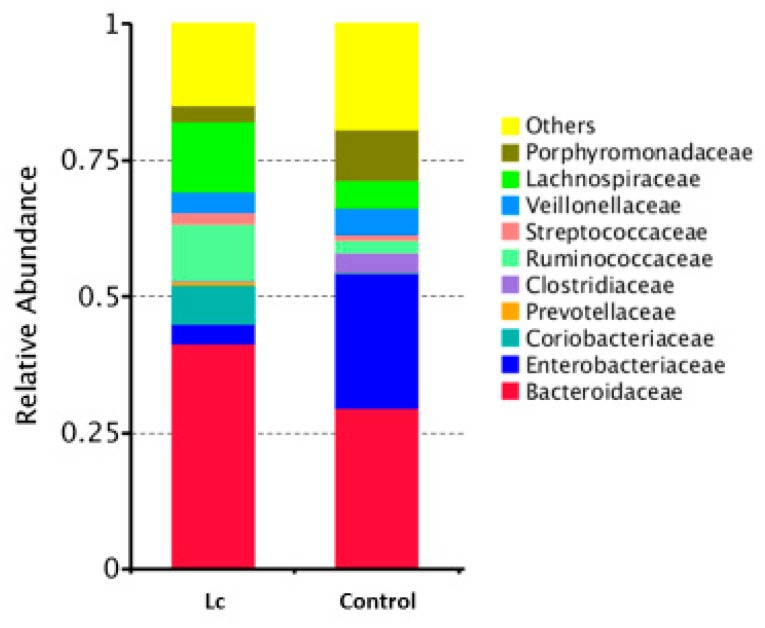
Differences in gut microbial composition between children with the probiotic *Lactobacillus casei* variety *rhamnosus* (Lc) supplement and the control group. Fecal samples of patients were collected at one week after enrollment in this study. Each color in this bar graph represents the corresponding taxon group at the family level in the legend.

**Figure 4:**
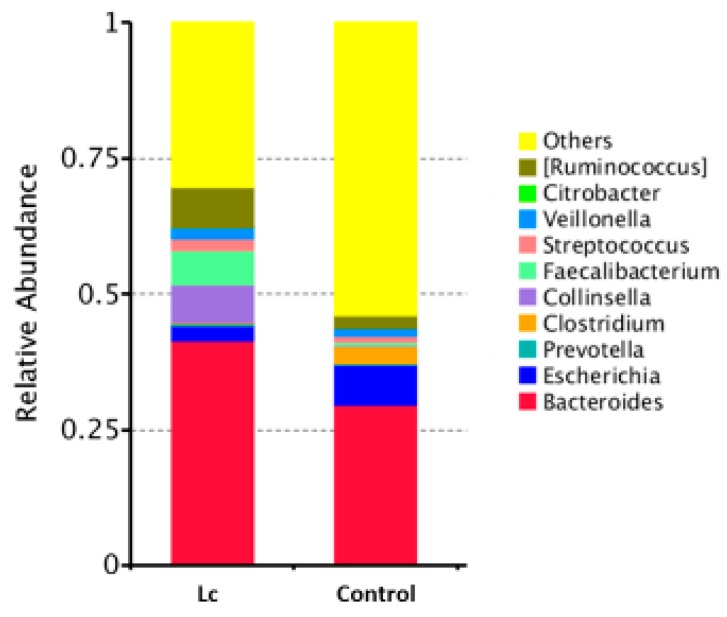
Differences in gut microbial composition between children with the probiotic *Lactobacillus casei* variety *rhamnosus* (Lc) supplement and the control group. Fecal samples of patients were collected at one week after enrollment in this study. Each color in this bar graph represents the corresponding taxon group at the genus level in the legend.

Alterations in the gut microbiota in children with the probiotic supplement: The relative abundance in gut microbial composition between children with or without the probiotic supplement was different. If there was daily supplementation with probiotic *Lactobacillus casei*, the relative abundance was higher in Bacteroidaceae, Ruminococaceae, Lachnospiraceae (family), and Bacteroides, Ruminoccus (genus), but lower in Enterobacteriaceae (family) and Escherichia, Clostridium (genus) (Figure 3 and Figure 4).

### 3.8. Fecal Immunoglobulin A

**Figure 5:**
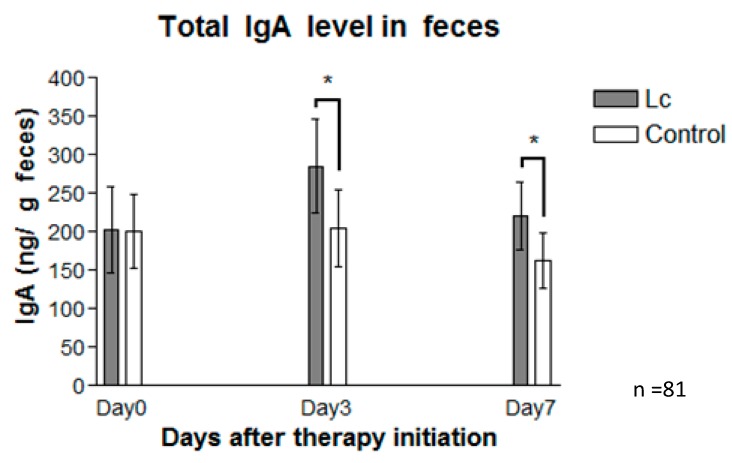
IgA values (described as ng/g feces) from fecal samples of the probiotic group (gray bar), and the control group (white bar) collected on Days 0, 3, and 7 after therapy was initiated. Fecal samples of patients prescribed with probiotics (*Lactobacillus casei* variety *rhamnosus*) produced a significantly higher level of total IgA than those of the control (placebo). The data are represented as the mean ± SEM (*n* = 81), and statistically significant differences compared with the control group; * *p* < 0.05.

Fecal total IgA levels were also measured on Days 0, 3, and 7. The mean ± standard deviation (SD) of fecal IgA concentration on Day 3 was 285.5 ± 60.9 ng/g in patients with the *Lactobacillus* supplement, and 205.7 ± 49.8 ng/g in the control group (Figure 1). Concentration of fecal IgA on Day 7 was 220.2 ± 43.8 ng/g in patients with the *Lactobacillus* supplement, and 162.8 ± 35.9ng/g in the control group. Figure 5 shows that the total IgA levels in the fecal extracts of the probiotic group were higher than those in the control, reaching statistical significance (*p* < 0.05). Analysis of such variance indicates that probiotics may promote a protective host intestinal immune response.

### 3.9. Measurement of Inflammatory Markers in Fecal Samples

#### 3.9.1. Fecal Lactoferrin

**Figure 6:**
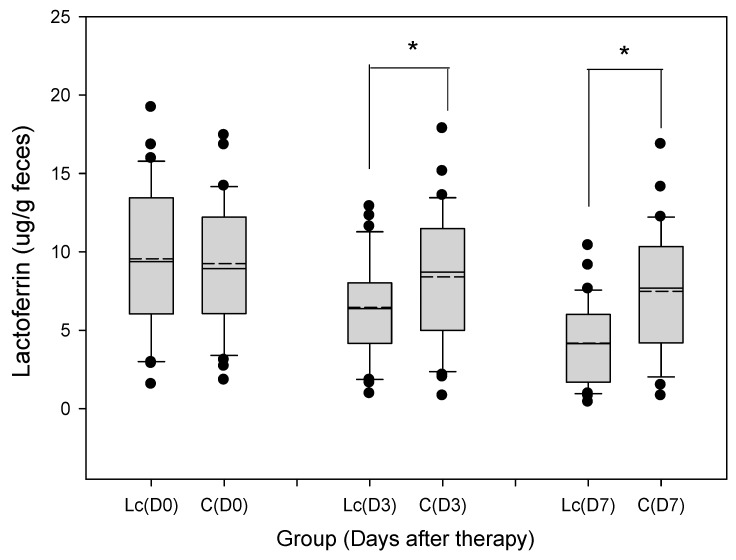
Grouped samples of fecal lactoferrin (μg/g feces) in children prescribed with probiotics (*Lactobacillus casei* variety *rhamnosus*) and the control (placebo). The figure shows the median, 10th, 25th, 75th, and 90th percentiles as vertical boxes with error bars. The remaining dots represent outliers less than the 10th or more than the 90th percentile. The fecal lactoferrin level was lower in patients with probiotic (*Lactobacillus casei* variety *rhamnosus*) consumption than those of the control (placebo) on Days 3 and 7 (horizontal line: Median; short dashed line: Mean; * *p* < 0.05).

The median and range of fecal lactoferrin concentration on Day 3 were 6.27 (1.16–12.95) μg/g in patients with the *Lactobacillus* supplement, and 8.78 (1.07–17.98) μg/g in the control group (Figure 6). The concentration of fecal lactoferrin on Day 7 was 4.10 (0.55–10.56) μg/g in patients with the *Lactobacillus* supplement, and 7.66 (0.89–16.94) μg/g in the control group. Fecal lactoferrin levels for patients with the probiotic supplement were significantly downregulated compared with those of the control. *p*-values for lactoferrin were each <0.05.

#### 3.9.2. Fecal Calprotectin

**Figure 7:**
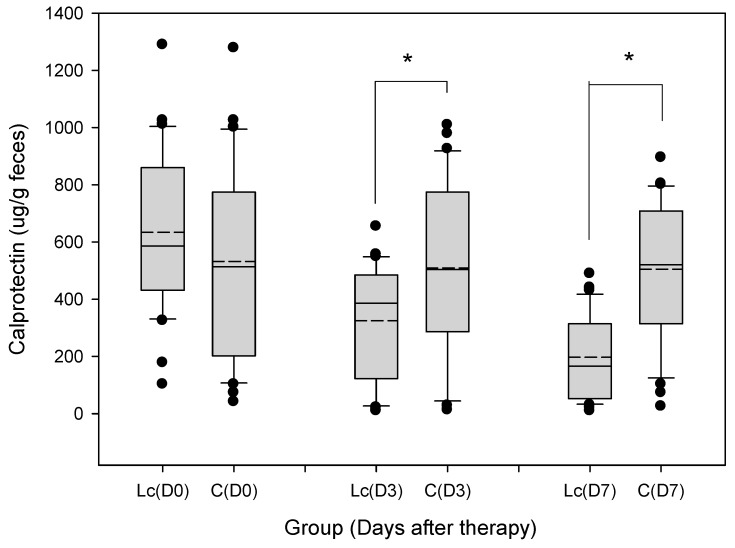
Grouped samples of fecal calprotectin (μg/g feces) in children prescribed with probiotics (*Lactobacillus casei* variety *rhamnosus*) and the control. The figure shows the median, 10th, 25th, 75th, and 90th percentiles as vertical boxes with error bars. The remaining dots represent outliers less than the 10th or more than the 90th percentile. Fecal calprotectin levels were lower in patients with probiotic (*Lactobacillus casei* variety *rhamnosus)* consumption than those of the control on Days 3 and 7 (horizontal line: Median; short dash line: Mean; * *p* < 0.05).

The median and range of fecal calprotectin concentration on Day 3 were 383 (25–661) μg/g in patients with the *Lactobacillus* supplement, and 513 (26–1019) μg/g in the control group (Figure 7). Concentration of fecal calprotectin on Day 7 was 164 (21–504) μg/g in patients with the *Lactobacillus* supplement, and 517 (30–898) μg/g in the control group. Fecal calprotectin levels for patients with the probiotic supplement were significantly downregulated compared with those of the control. *p*-values for calprotectin were each <0.05.

## 4. Discussion

Acute diarrhea is a major cause of childhood morbidity, with an emotional and economic burden for the families of affected children, as well as a burden on society [20]. Several types of medications, such as those affecting intestinal motility, ion transport, and adsorptive moieties, as well as living bacteria have been used in an attempt to reduce the duration of acute diarrhea [21]. Our study assessed the clinical effectiveness and fecal parameters of a commercially available probiotic product.

In our study, probiotics can reduce the severity of diarrhea with statistical significance (*p* < 0.05). Our findings were consistent with previous studies showing probiotics to be effective in reducing the duration of diarrhea [22,23,24,25]. Moreover, bowel habits improved under probiotic colonization. Probiotics also reduced abdominal pain or colic accompanying diarrhea episodes after the start of the treatment, as well as bloating or abdominal distension, especially on the day 7 and day 14 follow-ups. In our study, parents of children that were given probiotics seemed more satisfied with the better appetite and oral intake on the daily record, and even better weight gain.

However, some studies [26,27] found no positive effect of *Lactobacillus casei* on the clinical course of diarrhea, such as antibiotic-associated diarrhea. The possible reasons of why probiotics failed to do so are that the dosage, species of probiotics, or antibiotics therapy may reduce the anaerobic bacteria of the intestinal flora. In a recent report [28], compared to spontaneous post antibiotic recovery, a probiotic mixture containing at least 2.5 × 10^10^ active bacteria induced a delayed and persistently incomplete indigenous stool/mucosal microbiome reconstitution. Probiotics may also negatively influence the recolonization of commensal bacteria in antibiotics given to subjects [28,29].

Prominent differences were also observed in the diversity and composition of the gut microbiota of responders versus non-responders in immunotherapy for melanoma [30,31]. Moreover, some challenges remain to be solved concerning how to regulate gut microbiota to enhance the efficacy of cancer immunotherapy. Which specific composition of the gut microbiota is most conducive to ameliorating an anti-tumor immune response is unclear, and there are a variety of management options that alter gut microbiota [32,33]. The use of probiotics and antibiotics requires careful consideration. These long-term limitations of probiotics use should be addressed in specific situations, such as immunodeficiency individuals, patients undergoing anti-tumor therapy, or patients receiving a long course of antibiotics therapy.

Children usually suffer pain when collecting a blood sample through venipuncture or investigating with invasive endoscopy techniques. Noninvasive tests such as those involving stool samples are highly recommended for children and can help avoid pain. We; therefore, collected fecal samples and performed further analysis of bacteria counts and other inflammatory mediators. Previous studies showed that supplementation with *Lactobacillus* or *Bifidobacterium* species [34,35] restores the total *Lactobacillus* or *Bifidobacterium* counts in feces, respectively. In our study, probiotic preparations (*Lactobacillus casei* variety *rhamnosus*) may promote the major population of *Lactobacillus* in stool output, as well as *Bifidobacterium* counts in feces.

*Ruminococcus* is a Gram-positive anaerobic gut microbial genus in the Clostridia class and Ruminococcaceae family. Lachnospiraceae and Ruminococcaceae are two of the most abundant families from the Clostridiales order found in the mammalian gut environment, and they have been associated with the maintenance of intestinal health [36]. Lachnospiraceae such as *Eubacterium rectale*, *Eubacterium ventriosum*, *Coprococcus* spp., and *Roseburia* spp. have been associated with the production of butyrate, which is the major energy source of colonic epithelial tissue and may be involved in immune regulation and intestinal-barrier function [37,38,39,40]. In contrast, recent studies [41,42] have shown that butyrate is not universally beneficial for intestinal health. Lachnospiraceae and Ruminococcaceae differ with respect to their average numbers of carbohydrate-active genes and gene families, particularly glycoside hydrolases and carbohydrate-binding modules, which are more abundant and more diverse in Lachnospiraceae and Ruminococcaceae [43]. Our results revealed a higher abundance of Ruminococcaceae and Lachnospiraceae in children with the probiotic supplement.

Primary fermenters, such as *Bacteroides*, are gateways through which carbohydrates enter the network of metabolic interactions. *Bacteroides* degrade complex carbohydrates to their component monosaccharides, which, in turn, are metabolized through the sequential actions of metabolic pathways. The main excreted products of *Bacteroides* are acetate, propionate, and hydrogen [44,45,46]. Acetate, propionate, and butyrate are end-products of microbial action on endogenous and exogenous dietary and host-derived components. The protective effect of commensal bacteria may be regulated by the above metabolic products. Our results revealed a higher abundance of *Bacteroides* in children with the probiotic supplement.

Enterobacteriaceae are a prominent family of Gram-negative bacteria, which belong to the Enterobacteriales order and the Proteobacteria phylum. Enterobacteriaceae include many familiar pathogens, such as *Shigella, Salmonella*, *Escherichia coli*, *Klebsiella*, and *Yersinia*. Other disease-causing bacteria in this family include *Proteus, Serratia, Enterobacter*, and *Citrobacter* [46,47,48]. Our results revealed a lower relative abundance of Enterobacteriaceae in children with the probiotic supplement.

The function of fecal IgA is to agglutinate micro-organisms and to prevent the adherence of pathogenic microorganisms to the mucosal surface, as well as maintenance of intestinal microbial homeostasis [49,50,51]. There is a close interaction between the composition of intestinal micro-organisms and IgA production. Simultaneously, the continuous presence of commensal bacteria (e.g., probiotics) favors a constant stimulation of the immune system without invoking inflammatory responses [52]. In our study, total IgA levels in the fecal extracts of the *Lactobacillus* group were higher (*p* < 0.05) on Days 3 and 7 after therapy initiation than those in the control group. This result is a remarkable finding in probiotics treating acute childhood diarrhea, indicating that probiotics may enhance the protective immune response in the intestinal tract of the host.

Cells of the innate immune system secrete various enzymes and metabolites, including myeloperoxidase and lactoferrin, produced by activated neutrophils. Lactoferrin is mainly found in the oral cavity and intestinal tract where it can come into direct contact with pathogens such as viruses and bacteria. The most significant function of lactoferrin in mucosal defense is its antimicrobial activity. Lactoferrin can also amplify the actions of lysozyme and secretory immunoglobulin A [53]. Noninvasive fecal lactoferrin may prove useful in screening for inflammatory intestine activity in patients with abdominal pain and diarrhea [54,55]. According to our study, fecal lactoferrin levels were lower in patients with the probiotic treatment, indicating that probiotics attenuate inflammatory activity in the intestinal tract of the host.

A remarkable intestinal infection is the intense infiltration of neutrophils, macrophages, lymphocytes, and other inflammatory cells in the epithelial lining and the lamina propria of the colonic mucosa [56]. Neutrophils have been shown to be involved in the perpetuation of gut inflammation in acute infections caused by bacteria [8]. Fecal calprotectin, in particular, has for a long time been regarded as a promising marker of gastrointestinal pathology [57]. Fecal calprotectin has been proposed as a useful marker of gastrointestinal inflammation [19]. There are some studies that have explored the role of fecal calprotectin in acute infectious diarrhea. One study [58] enrolled children of less than five years of age. The concentration of fecal calprotectin correlates with intestinal inflammation, and children with bacterial gastroenteritis were found to have higher fecal calprotectin compared to viral gastroenteritis. Another study [59] investigated adult diarrhea and showed higher levels of calprotectin in bacterial infections. In our study, fecal calprotectin levels were lower in patients with probiotic treatment compared to those in the control. The above finding suggests that probiotics downregulate inflammatory activity in the intestinal tract of the host.

## 5. Conclusions

The efficacy of probiotic preparations for the treatment of acute childhood diarrhea is related to individual bacteria strains. Probiotic preparations (*Lactobacillus casei* variety *rhamnosus*) attenuated the severity of acute diarrhea in hospitalized children. They could restore the population of intestinal probiotic bacteria, modulate gut microbiota, and enhance secretary Immunoglobulin A levels with the reduction of intestinal inflammatory reactions, such as fecal lactoferrin and calprotectin.

## Figures and Tables

**Table 1:** Baseline characteristics of patients who completed the study.

Variables	Probiotics Group (*n* = 42)	Control Group (*n* = 39)	*p*-Value
**Age at entry (months)**	27.9 ± 15.6	27.6 ± 16.1	0.672
**Male/Female**	24:18	22:17	0.418
**Duration of diarrhea before intervention (h)**	37.3 ± 22.9	38.1 ± 21.8	0.621
**Vomiting**	13 (30.9%)	12 (30.7%)	0.868
**Fever on admission (>38.5 °C)**	20 (47.6%)	19 (48.7%)	0.514
**White blood cells (WBC) counts (10^6^ cells/L)**	12,354 ± 2865	12,075 ± 2742	0.426
**Abdominal pain/irritable crying on admission**	14 (33.3%)	13 (33.3%)	0.563

**Table 2:** Comparison of clinical situation during the intervention period between the probiotic *Lactobacillus casei* variety *rhamnosus* (Lc) group (*n* = 42) and the control group (*n* = 39).

Parameter	Day 0	Day 3	Day 7	Day 14
**Appetite/intake (score) #**				
Lc group	1.2 ± 0.4	* 3.5 ± 0.7	*4.1 ± 0.7	* 4.3 ± 0.6
Control group	1.2 ± 0.4	* 2.8 ± 0.6	*3.1 ± 0.6	* 3.8 ± 0.5
**Bloating/abdominal distension, *n* (%)**				
Lc group	20 (47.6%)	12 (28.6%)	* 6 (14.3%)	* 2 (4.8%)
Control group	17 (43.6%)	13 (33.3%)	* 11 (28.2%)	* 6 (15.4%)
**Abdominal pain/colic, *n* (%)**				
Lc group	17 (40.5%)	* 8 (19.0%)	* 4 (9.5%)	* 2 (4.8%)
Control group	16 (41.0%)	* 12 (30.8%)	* 9 (23.1%)	* 7 (17.9%)
**Diarrhea, *n* (%)**				
Lc group	42 (100.0%)	*29 (69.0%)	*8 (19.0%)	*3 (7.1%)
Control group	39 (100.0%)	*33 (84.6%)	*16 (41.0%)	*6 (15.4%)
**Vomiting, *n* (%)**				
Lc group	20 (47.6%)	6 (14.3%)	3 (7.1%)	2 (4.8%)
Control group	18 (46.2%)	7 (17.9%)	4 (10.3%)	3 (7.7%)
**Fever, *n* (%)**				
Lc group	29 (69.0%)	13 (31.0%)	1 (2.4%)	0 (0.0%)
Control group	26 (66.7%)	13 (33.3%)	1 (2.6%)	0 (0.0%)

# Score evaluated by family/parents (range 0~5),* *p* < 0.05. the bold format: significant variation.

**Table 3:** Alternation of fecal bacterial counts during childhood diarrhea. Lc group (*n* = 42) and control group (*n* = 39).

Fecal Bacteria log_10_ CFU/g of Stool	Day 0		Day 3		Day 7	
	Lc	Control	Lc	Control	Lc	Control
***Lactobacilli***	8.2 (7.6–9.0)	8.3 (7.6–9.1)	* 8.6 (8.0–9.4)	* 8.1 (7.4–8.7)	* 9.1 (8.3–9.8)	* 8.2 (7.6–8.8)
***Bifidobacterium***	9.3 (8.5–10.1)	9.4 (8.6–10.2)	9.5 (8.8–10.2)	9.1 (8.5–9.7)	* 9.7 (9.1–10.4)	* 9.0 (8.4–9.6)
**Gram-negative bacilli**	9.4 (8.8–9.7)	9.3 (8.7–9.6)	9.1 (8.6–9.5)	9.4 (8.8–9.7)	8.9 (8.4–9.2)	9.3 (8.8–9.6)
**Anaerobic bacteria**	9.5 (9.0–9.8)	9.5 (9.1–9.8)	9.4 (9.0–9.7)	9.5 (9.0–9.8)	9.4 (9.0–9.7)	9.5 (9.1–9.8)

Median of bacterial counts in fecal samples (95% confidence interval), Mann–Whitney *U* test. * *p* < 0.05. the bold format: significant variation.

## Abbreviations

Lc, *Lactobacillus casei* variety *rhamnosus*; CFU, colony-forming units; CRP, C-reactive protein; PBS, phosphate-buffered saline; EDTA, ethylenediaminetetraacetic acid; ELISA, enzyme-linked immunosorbent assay; IgA, immunoglobulin A; IBD, inflammatory bowel disease.
